# Proliferative vasculopathy and hydranencephaly–hydrocephaly syndrome or Fowler syndrome: Report of a family and insight into the disease's mechanism

**DOI:** 10.1002/mgg3.376

**Published:** 2018-03-03

**Authors:** Francesca Clementina Radio, Lavinia Di Meglio, Emanuele Agolini, Emanuele Bellacchio, Martina Rinelli, Paolo Toscano, Renata Boldrini, Antonio Novelli, Aniello Di Meglio, Bruno Dallapiccola

**Affiliations:** ^1^ Genetics and Rare Diseases Research Area Bambino Gesù Children's Hospital IRCCS Rome Italy; ^2^ Federico II University Naples Italy; ^3^ Ultrasound and Prenal Diagnosis “Aniello Di Meglio” Naples Italy; ^4^ Laboratory of Medical Genetics Bambino Gesù Children's Hospital IRCCS Rome Italy; ^5^ Pathology Unit Bambino Gesù Children's Hospital IRCCS Rome Italy

**Keywords:** *FLVCR2*, Fowler syndrome, hydrocephaly, multiple pterygium

## Abstract

**Background:**

Fowler syndrome is a rare autosomal recessive disorder characterized by hydranencephaly–hydrocephaly and multiple pterygium due to fetal akinesia. To date, around 45 cases from 27 families have been reported, and the pathogenic bi‐allelic mutations in *FLVCR2* gene described in 15 families. The pathogenesis of this condition has not been fully elucidated so far.

**Methods:**

We report on an additional family with two affected fetuses carrying a novel homozygous mutation in *FLVCR2* gene, and describe the impact of known mutants on the protein structural and functional impairment.

**Results:**

The present report confirms the genetic homogeneity of Fowler syndrome and describes a new *FLVCR2* mutation affecting the protein function. The structural analysis of the present and previously published *FLVCR2* mutations supports the hypothesis of a reduced heme import as the underlying disease's mechanism due to the stabilization of the occluded conformation or a protein misfolding.

**Conclusion:**

Our data suggest the hypothesis of heme deficiency as the major pathogenic mechanism of Fowler syndrome.

## 1 INTRODUCTION

Proliferative vasculopathy and hydranencephaly–hydrocephaly syndrome (PVHH, OMIM#225790), also known as Fowler syndrome, is a rare autosomal recessive disorder characterized by hydranencephaly–hydrocephaly, multiple pterygium due to fetal akinesia, a distinctive glomerular vasculopathy in the central nervous system (CNS) and retina, and diffuse ischemic lesions of the brain stem, basal ganglia, and spinal cord with calcifications. This disease was first described in 1972 in five affected fetuses from a single family, displaying “bubble‐like” cerebral hemispheres associated with the peculiar vasculopathy in CNS and retina (Fowler, Dow, White, & Greer, 1972). Subsequent reports have shown that hydranencephaly–hydrocephaly resulted from the destruction of brain tissue due to vascular anomalies, while other features were likely secondary to CNS injuries. The disorder is almost invariably prenatally lethal, usually detected by ultrasound between the 13 and 27 weeks of gestation (Williams et al., 2010). Only a single brother–sister pair has been reported, who survived beyond the neonatal period (Kvarnung et al., 2016).

The clinical diagnosis of PVHH syndrome has been molecularly confirmed in 15 families showing bi‐allelic mutations in *FLVCR2* gene (OMIM*610865). However, the pathogenesis of this disorder remains uncharacterized.

FLVCR2 is a cell surface protein, related to the heme exporter/retroviral receptor FLVCR1, functioning as a receptor for FY981 feline leukemia virus. The role of FLVCR2 was considered related to calcium metabolism (Brasier et al., 2004) and heme import (Duffy et al., 2010). The protein is expressed in a broad range of human tissues, including liver, placenta, kidney, and strongly in the brain (Duffy et al., 2010). Heme (iron and protoporphyrin IX) plays a pivotal role in many cellular processes accounting for a major component of hemoproteins (e.g., cytochromes, nitric oxide synthase, hemoglobin, etc.). Heme deficiency impacts on the mitochondrial respiratory chain, specifically onto the assembly of complex IV in human fibroblasts (Atamna, Liu, & Ames, 2001). Abnormalities of FLVCR2 function could affect this process with effects on neuronal migration dependent on oxidative phosphorylation, causing neurodegeneration and developmental abnormalities such as hydranencephaly–hydrocephaly (Castro‐Gago, Eirís‐Puñal, & Iglesias‐Diz, 1999). To date, it remains unclear whether the proliferative vasculopathy observed in PVHH syndrome is primary or secondary to neurodegeneration.

Here, we report on two fetuses affected by Fowler syndrome in which the diagnosis was suggested, after the second pregnancy's termination, based on pictures taken before autopsy and corroborated by X‐rays features and targeted genetic testing.

## 2 MATERIAL AND METHODS

### 2.1 Ethical compliance

The research was conducted in accordance with the Declaration of Helsinki and approved by the local Ethics Committee.

### 2.2 Case report

**Figure 1:**
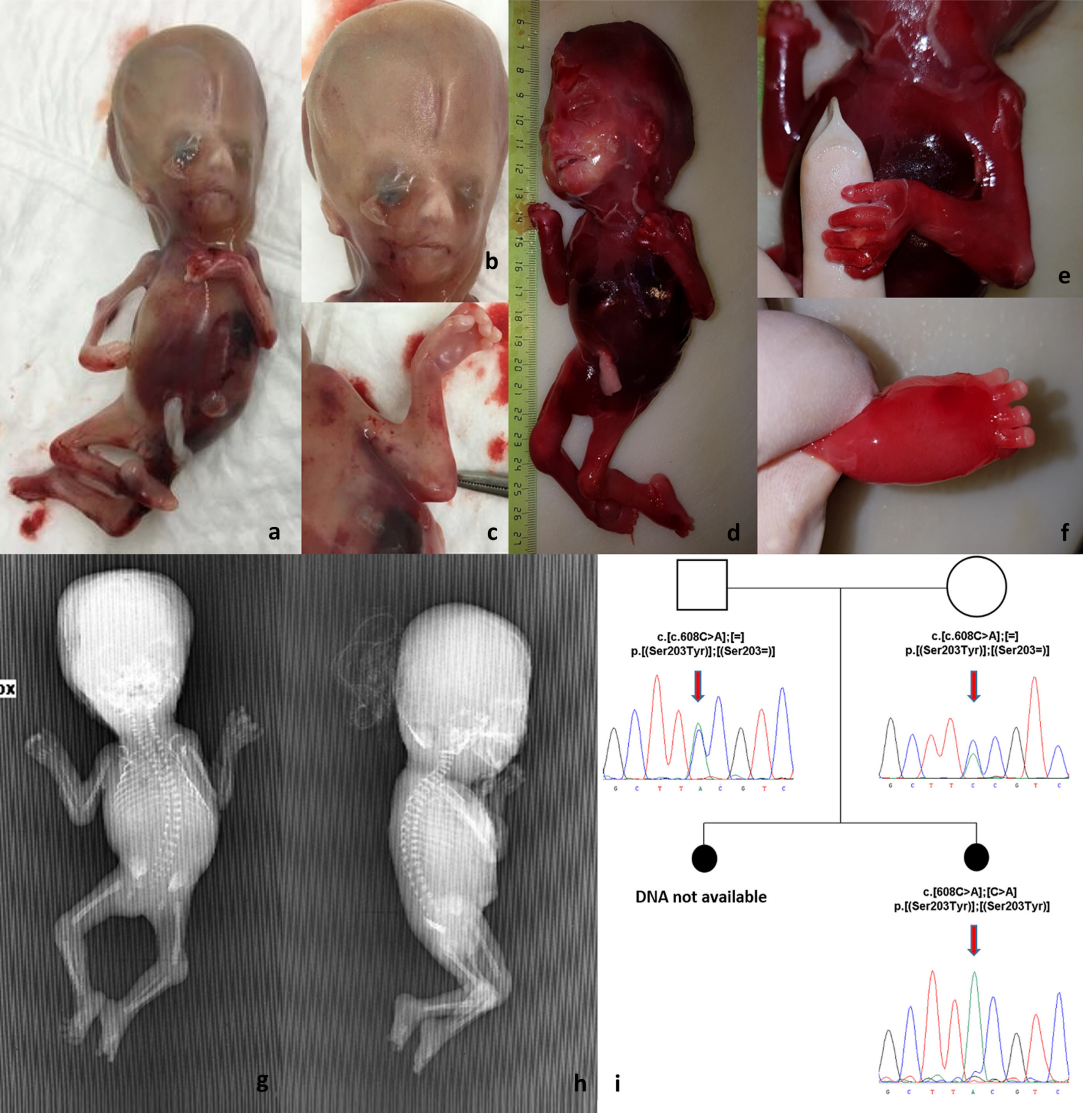
(a–c) First fetus: (a) clinical features; (b) macrocephaly and craniofacial abnormalities; (c) pterigium of the elbow. (d–h) Second fetus: (d) clinical features; (e) pterigium of the elbow; (f) edema of feet; (g–h) scoliosis and sacrum agenesis. (i) Family's pedigree and electropherograms of *FLVCR2* mutation (NM_017791.2)

The healthy parents were 28 years old at time of first conception. In the first pregnancy, prenatal ultrasound at 17 weeks of gestation disclosed a single fetus with hypotelorism, hydranencephaly, absent visualization of Willis polygon, and defective cerebral cortical development. Bilateral hydrothorax and generalized edema were also present. Kidneys were small and dysplastic with a depleted bladder. Based on these defects, the informed parents opted for pregnancy's termination. The macroscopic post‐mortem evaluation revealed multiple pterygia, arthrogryposis/camptodactyly, and abnormalities of the craniofacial appearance related to the CNS anomalies (Figure 1a–c). No microscopic or radiological investigation was performed and no biological specimen was collected. Genetic counseling was not offered to parents. In the second pregnancy, 6 months later, prenatal ultrasounds at 13 and 16 weeks were unremarkable. An additional ultrasound scan at 20 weeks disclosed hydranencephaly with visualization of the cerebral falx and vascular structure of the Willis polygon, and without visualization of cerebral cortical and posterior fossa structures. Fetal akinesia deformation sequence was suspected. Post‐mortem evaluation disclosed a clinical spectrum of defects overlapping those of the first fetus (Figure 1d–f). X‐ray imaging showed scoliosis and sacrum agenesis (Figure 1g–h). The microscopic evaluation disclosed atrophic cortical mantle and glomeruloid vascular structures disseminated in the spinal cord, cerebral system, and cerebellum. Large necrotic areas and calcifications were also detected.

Following the second termination, parents requested genetic counseling. Based on recurrence in two subsequent pregnancies of fetal akinesia deformation sequence with hydranencephaly and a distinct CNS vasculopathy, a diagnosis of Fowler syndrome was suggested and targeted genetic testing performed on tissues sampled from the second fetus and on parental blood.

### 2.3 Molecular analysis

The clinical diagnosis was molecularly confirmed by Sanger sequencing of *FLVCR2* gene on a DNA sample extracted by the brain of the second fetus that disclosed a homozygous missense mutation NM_017791.2:c.608C>A (p.Ser203Tyr). Segregation analysis confirmed that parents were heterozygous carriers (Figure 1i; primer sequence and PCR conditions were available upon request). The identified variant has not been reported in the literature or public databases suggesting a possible consanguinity of the parents supported by a high rate of shared rare and very rare variants spanning the entire genome (data not shown).

### 2.4 Structural analysis

Structural analysis was carried out to evaluate the impact of the present and published *FLVCR2* gene mutations found in PVHH affected subjects on the protein function. Structurally characterized homologues of the human Feline leukemia virus subgroup C receptor‐related protein 2 (FLVCR2) were found with RaptorX (Källberg et al., 2012) and FLVCR2 homology models were built on the top scoring five targets. Prediction of membrane protein topology was made with TOPCONS (Tsirigos, Peters, Shu, Käll, & Elofsson, 2015). Molecular graphics were rendered with PyMOL (http://www.pymol.org/).

## 3 RESULTS AND DISCUSSION

To date about 45 fetuses affected by Fowler syndrome from 27 families have been reported. In 2010, next‐generation sequencing technologies led to the identification of the molecular defect underlying this disorder (Lalonde et al., 2010); (Meyer et al., 2010); (Thomas et al., 2010). The present report confirms the genetic homogeneity of Fowler syndrome and describes a new *FLVCR2* mutation affecting the protein function.

FLVCR2 belongs to the major facilitator superfamily (MFS), and represents the largest group of secondary active membrane transporters, whose members are specialized in the permeation of a variety of molecules. FLVCR2 has 12 predicted transmembrane helices, and works as a heme importer (Duffy et al., 2010) and likely as a calcium transporter (Brasier et al., 2004). MFS proteins are thought to transport substrates using a rocker‐switch mechanism (Huang, Lemieux, Song, Auer, & Wang, 2003) involving two major conformations, inward and outward, achievable by mutual rotation of ca. 40 degree of the two homologous halves of the protein (TM 1‐6 and 7‐12 for MFS proteins with 12 TMs) forming the transmembrane core.

**Figure 2:**
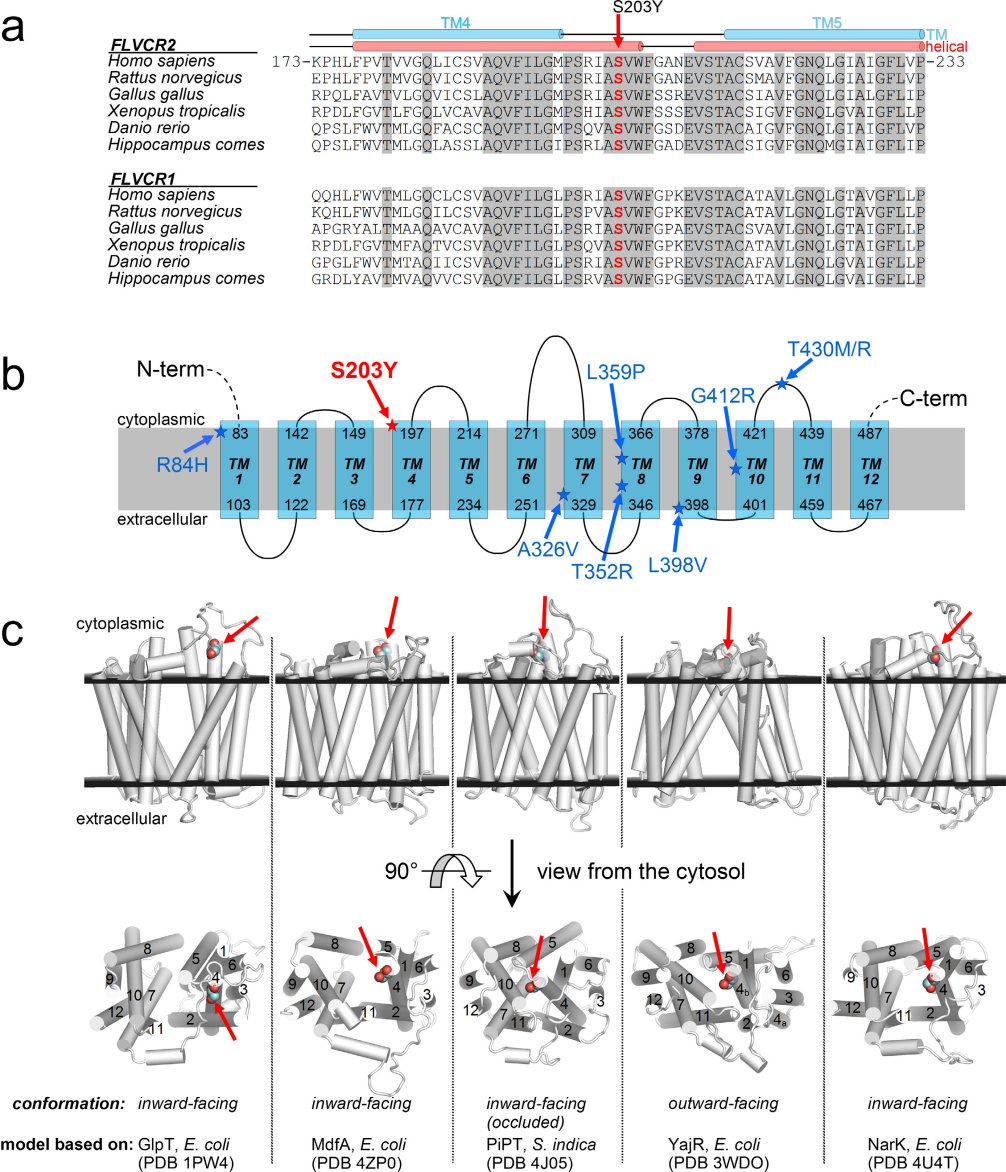
(a) Multiple sequence alignment of FLVCR2 protein (and the FLVCR1 paralogue) among species around the sites of the p.Ser203Tyr mutation (invariant residues are grayed). (b) Scheme of predicted membrane protein topology of FLVCR2. In red, the amino acid change identified in the present family. In blue, the amino acid change identified to date in Fowler syndrome. (c) Homology models of FLVCR2 (residues 86–491) based on structurally characterized MFS transporters. In the upper row of structures (viewed from the membrane side), the extracellular and cytoplasmic membrane layers (black planes) are positioned according to the predicted TM topology. The lower row represents the above structures rotated by 90 degree and viewed from the cytosol (TM regions 1–12 are labeled). Ser203 (site of the p.Ser203Tyr mutation) is shown as colored spheres and it is marked by red arrows. Substrates are transported across the membrane through the central part of the protein along the direction perpendicular to the figure plane of the cytosol view

**Figure 3:**
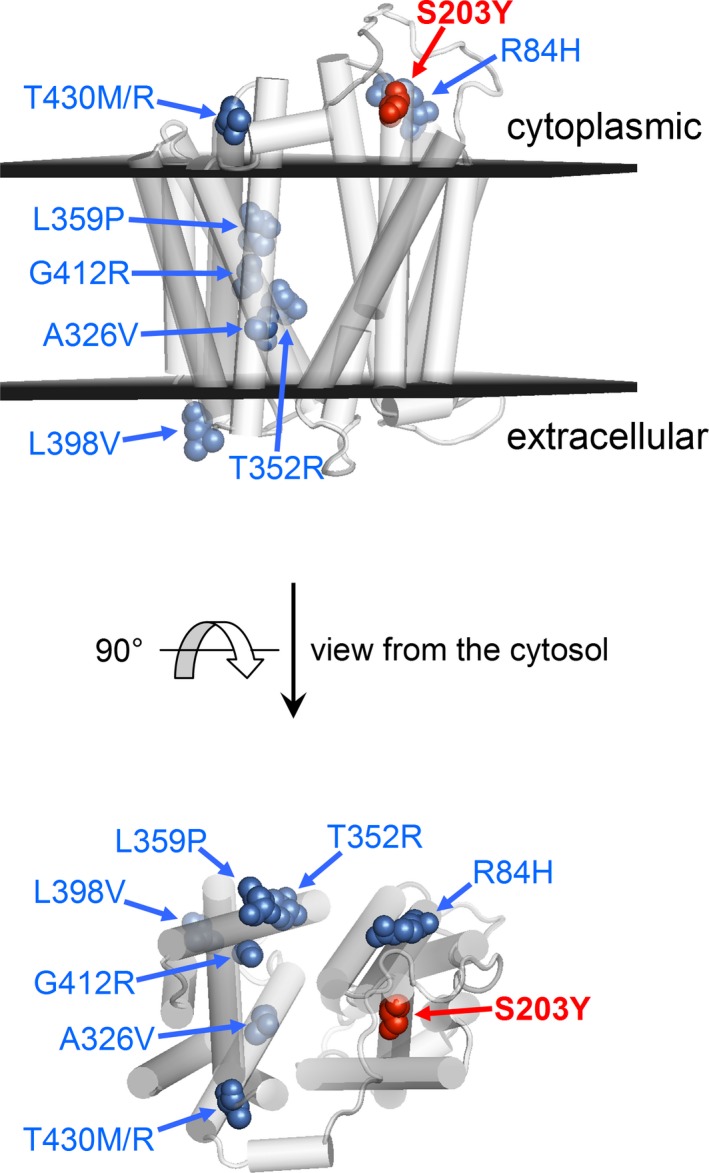
Homology models of FLVCR2 (residues 86–491) based on GlpT di *E. coli*, PDB 1PW4 model (first on the left in Figure 2c). Known mutants are shown as colored spheres and marked by blue arrows. Substrates are transported across the membrane through the central part of the protein along the direction perpendicular to the figure plane of the cytosol view

We have examined how the protein functioning might be affected by the p.Ser203Tyr amino acid change and whether it may be responsible for the observed phenotype. As shown in Figure 2a, the affected serine residue is invariant in the protein sequences of vertebrates for both FLVCR2 and the FLVCR1 paralogue, and predicted to be in the helical portion extending from the fourth transmembrane (TM) region into the cytosol (Figure 2a,b). Congruently with this prediction, mapping the site of the mutation on models obtained from structurally characterized MFS proteins allows to locate Ser203 in the helix protruding from the TM4 region into the cytosol (Figure 2c). Interestingly, this helix packs with the cytosolic helical extensions of TM10 and TM11 in addition to the covalently linked helix of TM5 in two FLVCR2 conformers: the model arranged in outward‐facing conformation (based on the *E. coli* YajR structure), and the model with inward‐facing and occluded conformation (constructed on the *S. indica* PiPT structure). These interhelix interactions occur at the level of Ser203. Thus, the p.Ser203Tyr amino acid change, which implies the replacement of a tiny serine with the much larger and aromatic tyrosine, introduces additional and undue interactions with surrounding residues, and causes an important impact on the stability of conformations, critical for the substrate binding and membrane transport ability of the FLVCR2 protein. These observations were inferred from the homology modeling of FLVCR2 with other MSF members with known 3D structure. As a matter of fact, by belonging to the Major Facilitator Superfamily, FLVCR2 should transport substrates through the same rocker‐switch mechanism proposed for this superfamily and undergo the same key conformational changes as captured in crystal structures of homologous MFS proteins (Figure 2c). Our data suggest the hypothesis of heme deficiency as the major pathogenic mechanism for this disorder. In fact, the protein affected by the p.Ser203Tyr amino acid change seems to maintain the occluded conformation with a reduced heme import. Interestingly, the *FLVCR2* missense mutations related with Fowler syndrome usually affect transmembrane domains with possible impact onto the channel proper function or folding, while additional null mutations seem to cause lack of protein, supporting the hypothesis of an impaired heme import as the principal pathogenic mechanism. The structural analysis of previously published *FLVCR2* mutations supports this hypothesis (Figure 3) due to the stabilization of the occluded conformation or a protein misfolding. *In vitro* and *in vivo* functional validation are needed to support this hypothesis.

## CONFLICT OF INTEREST

The authors declare no conflict of interest.

## ETHICAL APPROVAL

Ethics approval Ethical Committee of Bambino Gesù Children's Hospital.
